# Development and psychometric validation of a health literacy competency scale for nurses, and its application in a cross-sectional survey: a methodological study

**DOI:** 10.3389/fpubh.2026.1978033

**Published:** 2026-09-28

**Authors:** Xuemei Bao, Shuhan Liu, Xiaoxiao Hu, Qinghua Zhang, Mei Jiang, Yingting Gan, Yu Wang

**Affiliations:** 1 Chongqing Red Cross Hospital, Chongqing, China; 2 School of Medicine & Nursing Sciences, Huzhou Normal University, Huzhou, Zhejiang, China; 3 Huzhou Key Laboratory of Precise Prevention and Control of Major Chronic Diseases, Huzhou Normal University, Huzhou, Zhejiang, China

**Keywords:** competency, health literacy, influencing factors, nurse, psychometric validation, scale development

## Abstract

**Objectives:**

This study aimed to develop the Health Literacy Competencies Scale for Nurses (HLC-N) and examine the current status and influencing factors of health literacy competencies (HLC) among nurses in China.

**Design:**

A multi-phase methodological study consisting of three stages: (1) item generation (literature review, semi-structured interviews with 12 nurses, a two-round Delphi process with 16 experts, and pilot testing with 30 nurses); (2) psychometric validation (item analysis, exploratory factor analysis [EFA], confirmatory factor analysis [CFA], and reliability testing); and (3) a cross-sectional survey to examine current status and influencing factors.

**Setting:**

Psychometric validation was conducted across general hospitals in Chongqing, Sichuan, and Zhejiang provinces (July–August 2025). The cross-sectional survey was carried out in general hospitals across three districts of Chongqing, China (August–September 2025).

**Participants:**

Psychometric validation included 860 in-service nurses (467 for EFA; 393 for CFA and reliability; 81 for test–retest). Inclusion criteria: registered nurses with ≥1 year of clinical experience. Exclusion criteria: intern nurses, refresher nurses, or those who had previously participated in scale development. The cross-sectional survey included 647 nurses from primary, secondary, and tertiary hospitals in Chongqing.

**Results:**

The final HLC-N comprised 39 items across five dimensions, explaining 72.6% of the variance, with good model fit: χ^2^/df = 2.223, RMSEA = 0.056 (90% CI 0.052 to 0.060), CFI = 0.942. Cronbach‘s α for the total scale was 0.958 (95% CI 0.949 to 0.966). Among 647 nurses, the mean HLC score was 154.41 (SD 20.60). In multivariate regression, health status (β = 0.087, 95% CI 0.494 to 7.089, *p* = 0.024), health literacy training (*β* = 0.164, 95% CI 2.899 to 10.735, *p* = 0.001), and competency training (*β* = 0.122, 95% CI 1.101 to 8.940, *p* = 0.012) were independent predictors, explaining 7.8% of the variance (adjusted *R^2^* = 0.078).

**Conclusion:**

The HLC-N demonstrated robust psychometric properties and may serve as a reliable tool for assessing HLC among nurses in China. Targeted training and health promotion for nurses could enhance their HLC. Future research should explore additional predictors and validate the scale across diverse nursing populations.

## 1 Introduction

Health literacy (HL) is a core component of global public health strategy, and its importance has been widely recognized and emphasized in recent years (1). The World Health Organization (WHO) defines HL as the ability of individuals to access, understand, and use credible health information and services, which is essential for promoting the healthy development of individuals and communities (2). International charters and declarations, such as the *Bangkok Charter for Health Promotion* and the *Shanghai Declaration on the 2030 Agenda for Sustainable Development*, have consistently underscored the role of HL in disease prevention and improving health outcomes (3, 4).

Despite the growing emphasis on patient HL, the competency of healthcare providers—particularly nurses—to deliver precise health promotion tailored to patients with varying HL levels remains a critical yet underexplored issue in current nursing practice. Nurses, as frontline healthcare professionals who spend the most time with patients, play a pivotal role in health education and promotion (5). While nurses generally possess a certain level of personal HL, evidence suggests that personal HL does not automatically translate into the professional competency required to assess, communicate with, and educate patients who have limited or diverse HL abilities (6, 7). This disconnect may lead to ineffective health communication, poor patient adherence, and suboptimal health outcomes (8, 9).

### 1.1 Conceptual framework: the iceberg model

To address this gap, it is necessary to have a valid and reliable instrument to assess nurses’ health literacy competencies (HLC)—defined as the integrated knowledge, skills, attitudes, and collaborative abilities necessary to provide health promotion that is responsive to patients’ HL levels (10, 11). This study adopted the Iceberg Model (12) as its theoretical framework. The Iceberg Model distinguishes between surface-level observable competencies (e.g., skills, behaviors) and underlying, less visible attributes (e.g., knowledge, attitudes, motivation). This hierarchical structure aligns well with the multidimensional nature of HLC, which encompasses not only observable health education practices but also foundational HL knowledge, communication skills, inter-professional collaboration abilities, and intrinsic motivational drivers.

### 1.2 Gaps in existing instruments

Although several instruments exist for measuring HL among patients or the general population, tools specifically designed to evaluate HLC among nurses are scarce, particularly within the Chinese cultural context (13, 14). Existing tools often lack theoretical grounding, fail to capture the multidimensional nature of HLC (e.g., inter-professional collaboration, resource navigation), or have not been rigorously psychometrically tested in Chinese nurse populations.

First, some tools target a generalized “health professional” population rather than nurses specifically. For example, Chang et al. (13) validated the Instrument of Health Literacy Competencies (IOHLC) for Chinese-speaking health professionals. However, this instrument was developed based on data collected as early as 2012–2013 and focuses on a broad range of health professionals, failing to capture nurses’ unique roles in continuous health education throughout the entire patient care process (admission, hospitalization, discharge, and follow-up), as well as deeper elements such as emotional support, empathy, and trust-building in nurse–patient communication.

Second, some instruments focus on “health education ability” rather than HLC, resulting in conceptual misalignment. Mi et al. (14) developed a self-rating scale of health education competency for nursing staff, which demonstrated good reliability and validity. However, this scale does not encompass key components of HLC such as inter-professional collaboration, resource navigation, and intrinsic motivation to actively improve patients’ HL.

Third, existing tools often suffer from limited applicability across different care settings. Niu et al. (15) developed a health education ability assessment scale specifically for community nursing staff, while Feng X. et al. (16) developed an evaluation index system for dental nurses. Although these instruments have satisfactory psychometric properties, their applicability is restricted to specific clinical scenarios, lacking generalizability to nurses in various departments of general hospitals.

Fourth, the Chinese version of the IOHLC adapted by Qi (17) still targets a generalized “medical staff” population and does not specifically address nurses’ unique professional competencies. Moreover, this instrument fails to capture the continuity of nursing care and the intrinsic motivational aspects central to effective health literacy promotion.

Given these limitations, there is an urgent need to develop a nurse-specific, theoretically grounded, and psychometrically robust HLC assessment tool. Therefore, based on the Iceberg Model (12) and the previously adapted IOHLC (17), this study aimed to: (1) develop and evaluate the psychometric properties of the Health Literacy Competencies Scale for Nurses (HLC-N) tailored to the Chinese cultural context; and (2) investigate the current status and influencing factors of nurses’ HLC, providing empirical evidence for targeted training programs.

## 2 Materials and methods

### 2.1 Study design and phases

This study employed a multiphase, cross-sectional design for scale development and psychometric validation, followed by a cross-sectional survey to investigate the current status and influencing factors of nurses’ health literacy competencies (HLC). The research was conducted in three phases: (1) item generation and content validity (literature review, semi-structured interviews, Delphi expert consultation, pilot test); (2) psychometric evaluation (item analysis, exploratory factor analysis [EFA], confirmatory factor analysis [CFA], reliability testing); and (3) cross-sectional survey (current status and influencing factors).

### 2.2 Participants

Scale development and psychometric validation sample: A total of 860 in-service nurses were recruited from general hospitals in Chongqing, Sichuan, and Zhejiang provinces using convenience sampling. The minimum sample size for EFA was determined based on the recommendation that the participant-to-item ratio should be at least 5:1–10:1, with a minimum of 200 participants (18). Our sample of 467 for EFA exceeded this requirement. For CFA, a sample size of 200–300 is recommended (19); our sample of 393 met this criterion. Among the 860 nurses, 467 were included in item analysis and EFA, 393 in CFA and internal consistency reliability testing. Of these, 81 nurses were randomly selected from the total sample of 860 to complete a second assessment after a two-week interval for test–retest reliability.

Delphi expert sample: A panel of 16 experts was recruited for the two-round Delphi consultation. The inclusion criteria were: (a) bachelor’s degree or higher; (b) at least five years of relevant work experience; (c) intermediate professional title or above; and (d) research background in health literacy or competency, or experience in assessment tool development. The exclusion criterion was inability to return the completed questionnaire within the specified timeframe.

Pilot test sample: A pilot test was conducted with 30 nurses to assess the clarity and comprehensibility of the initial scale items.

Cross-sectional survey sample: For the cross-sectional survey investigating current status and influencing factors, 647 in-service nurses were recruited from general hospitals in Chongqing. Inclusion criteria were: (a) registered nurses; (b) at least one year of clinical work experience; (c) provided informed consent and volunteered to participate in this study; (d) employed in primary, secondary, or tertiary general hospitals. Exclusion criteria were: (a) intern nurses or refresher nurses; (b) those who had previously participated in the item screening or questionnaire completion of this scale.

### 2.3 Patient and public involvement

Patients and/or the public were not involved in this study because the research focused on developing a professional competency scale for in-service nurses, which required expert knowledge of nursing practice and health literacy education.

### 2.4 Sampling procedure

A combined sampling strategy was employed across two phases involving large-scale participant recruitment.

Phase 1 (Scale development – psychometric validation): A convenience sampling method was used to recruit 860 in-service nurses from general hospitals in Chongqing, Sichuan, and Zhejiang provinces between July 2025 and August 2025. The sample was divided into two subsets: 467 nurses for item analysis and EFA, and 393 nurses for CFA and internal consistency testing. Additionally, 81 nurses were randomly selected from the total sample to complete a second assessment after a two-week interval for test–retest reliability.

Phase 2 (Cross-sectional survey – current status and influencing factors): A multistage sampling method was applied. First, based on the *2023 Chongqing Statistical Yearbook* (20) stratified sampling was used to divide the 38 districts/counties of Chongqing into three strata (high, medium, low) according to geographical location and economic development level. Second, one district was randomly selected from each stratum using a random number table method. Third, all nurses from primary, secondary, and tertiary general hospitals within each selected district were recruited as a cluster sample to participate in the questionnaire survey. Data collection for Phase 2 was conducted between August 2025 and September 2025.

### 2.5 Measures

#### 2.5.1 General information questionnaire

A general information questionnaire was self-designed by the researchers to collect demographic and professional characteristics, including gender, age, professional title, hospital level, job category, years of work experience, clinical department, education level, employment type, personal monthly income, marital status, self-reported health status, and prior training experience in health literacy or competency enhancement.

#### 2.5.2 Health Literacy Competencies Scale for nurses (HLC-N)

The HLC-N was developed using a two-round modified Delphi technique with 16 experts. The final scale comprises 39 items across five dimensions: Health Literacy Knowledge (7 items), Health Education Communication Competency (8 items), Health Education Practice Competency (8 items), Inter-professional and Resource Collaboration Competency (10 items), and Attitude and Motivation (6 items). A 5-point Likert scale (1 = strongly disagree to 5 = strongly agree) is used, with two reverse-scored items (items 23 and 35). The total score ranges from 39 to 195 points, based on the percentile-based criterion (60 and 80% of the maximum score), scores were categorized into three levels: low (<117), moderate (117–156), and high (>156).

## 3 Data collection

Data collection was conducted between July 2025 and September 2025. Prior to the survey, trained research assistants explained the purpose and procedures of the study to potential participants. Written informed consent was obtained from all participants. Questionnaires were distributed either in paper-and-pencil format during regular work breaks or via an online platform depending on hospital preferences. For the test–retest reliability assessment, 81 nurses completed the HLC-N again after a two-week interval under identical conditions. All questionnaires were checked for completeness upon collection; those with missing data exceeding 5% or showing obvious response patterns were excluded from analysis.

## 4 Ethical approval

This study was conducted in accordance with the Declaration of Helsinki (revised in 2013). Ethical approval was obtained from the Ethics Committee of the Medical College of Huzhou Normal University (Approval No.: 202504-05). All participants were informed of the study‘s purpose, voluntary nature, and their right to withdraw at any time without negative consequences. Written informed consent was obtained from all participants prior to data collection. The study did not involve any invasive procedures or experimental interventions, and all data were anonymised and analysed confidentially to protect participant privacy.

## 5 Statistical analysis

Statistical analyses were performed using SPSS 25.0 and AMOS 29.0 (IBM Corp., Armonk, NY, USA). The psychometric properties of the HLC-N were evaluated in two phases: Phase 1 (*n* = 467) for item analysis and EFA, and Phase 2 (n = 393) for CFA and reliability testing. Test–retest reliability was assessed on the Phase 2 sample after a two-week interval.

### 5.1 Item analysis

Three methods were used for item analysis:

Critical ratio method: Total scale scores were sorted from high to low. The top 27% were classified as the high-score group and the bottom 27% as the low-score group. Independent-sample t-tests were used to compare differences between the two groups on each item. Items with a critical ratio (CR) < 3 or *p* > 0.05 were considered for deletion.

Correlation coefficient method: Pearson correlation coefficients between each item score and the total scale score were calculated. Items with non-significant correlations (*p* > 0.05) or correlation coefficients (r) < 0.4 were considered for deletion.

Homogeneity test: If the overall reliability coefficient (Cronbach’s α) increased after deleting a specific item, that item was considered for deletion (18).

### 5.2 Validity testing

Validity testing included content validity and construct validity.

Content validity: Experts familiar with the research field were invited to rate the items on the degree of congruence between each item and the measurement target. The content validity index (CVI) was used as the evaluation indicator, including the item-level CVI (I-CVI) and scale-level CVI (S-CVI). The criteria were I-CVI ≥ 0.78 and average S-CVI (S-CVI/Ave) ≥ 0.90 (21).

Construct validity – exploratory factor analysis: The Kaiser-Meyer-Olkin (KMO) test and Bartlett’s test of sphericity were used to determine whether the data were suitable for factor analysis. Generally, a KMO value > 0.8 and a significant Bartlett’s test (*p* < 0.05) were required. Principal component analysis with varimax orthogonal rotation was used for factor extraction. Factor extraction and item retention followed these criteria: (a) eigenvalue > 1; (b) cumulative variance contribution rate > 50%; (c) each factor containing at least two items; (d) factor loading > 0.5 for each item on one factor; items with loading > 0.5 on two or more factors and a loading difference < 0.2 were considered cross-loading items and were deleted; (e) combined judgment with scree plot results (18). Pearson correlation coefficients were used to analyze the correlations between factors and between each factor and the total score (22).

Construct validity – confirmatory factor analysis: Model fit was evaluated using the following criteria: χ^2^/df < 3, comparative fit index (CFI) > 0.90, incremental fit index (IFI) > 0.90, normed fit index (NFI) > 0.90, Tucker-Lewis index (TLI) > 0.90, and root mean square error of approximation (RMSEA) < 0.08 (22). The goodness-of-fit index (GFI) > 0.80 was considered acceptable. Convergent validity was evaluated using average variance extracted (AVE) and composite reliability (CR). The criteria for convergent validity were AVE > 0.5 and CR > 0.7 for each factor (19). Discriminant validity was established when the square root of AVE for each factor was greater than the absolute value of the correlation coefficient between any two factors (19).

### 5.3 Reliability testing

Internal consistency reliability: Cronbach’s α coefficient and split-half reliability were used to evaluate internal consistency. The criteria were Cronbach’s α > 0.8 for the total scale and > 0.6 for subscales (23).

Test–retest reliability: A test–retest reliability coefficient > 0.7 indicated good temporal stability of the scale (23).

### 5.4 Cross-sectional survey statistical analysis

For the cross-sectional survey (*n* = 647), statistical analyses were performed using SPSS 25.0. Descriptive statistics were presented as frequencies and percentages for categorical variables, and as mean ± standard deviation (x ± s) for continuous variables. Normality of data distribution was examined using skewness and kurtosis. Group differences were compared using independent sample t-tests and one-way analysis of variance (ANOVA). Multiple linear regression analysis (enter method) was used to identify influencing factors of HLC. A two-tailed *p* < 0.05 was considered statistically significant for all analyses.

## 6 Results

### 6.1 Part 1: scale development results

Based on literature review, five HLC assessment tools were identified (10, 15, 17, 24, 25). Two additional studies (26, 27) informed the motivation dimension of the scale. Semi-structured interviews with 12 nurses yielded four themes and nine sub-themes, leading to 7 new items and 2 dimension splits, resulting in a 53-item version. Two Delphi rounds with 16 experts (response rates: 100, 94.5%; authority coefficients >0.8; Kendall’s W: 0.217 → 0.355, *p* < 0.05) resulted in 23 deletions, 13 revisions, 12 additions, and 3 dimension mergers, yielding a 42-item scale. A pilot test with 30 nurses confirmed item clarity, and no further modifications were made.

### 6.2 Part 2: psychometric evaluation results

#### 6.2.1 General characteristics of the survey participants (psychometric sample)

The two-phase samples showed no statistically significant differences in key demographic variables including professional title, and training experience (*p* > 0.05), indicating good balance and comparability between the two samples on critical demographic characteristics (see Table 1).

**Table 1:** General characteristics of the survey participants (psychometric sample).

Characteristic	*N* = 467		*N* = 393		F/t	*p*
	*N*	%	*N*	%		
Professional title						
Junior	227	48.61	166	42.24	−1.789^b^	0.074
Intermediate	219	46.90	208	52.93		
Senior	21	4.50	19	4.83		
Education level						
Associate degree	97	20.77	47	11.96	11.88^a^	<0.001
Bachelor’s degree or above	370	79.23	346	88.04		
Marital status						
Unmarried	138	29.55	104	26.46	2.503^a^	0.480
Married	317	67.88	279	70.99		
Divorced	10	2.14	10	2.54		
Widowed	2	0.43	0	0.00		
Self-reported health status						
Good	324	69.38	269	68.45	0.459^a^	0.965
Fair	137	29.34	118	30.03		
Poor	5	1.07	5	1.27		
Other	1	0.21	1	0.25		
Received health literacy/health management training						
Yes	253	54.18	215	54.71	0.024^a^	0.876
No	214	45.82	178	45.29		
Received competency enhancement training						
Yes	237	50.75	206	52.42	0.238^a^	0.626
No	230	49.25	187	47.58		

^a^Chi-square test for categorical variables; ^b^Independent-sample *t*-test for continuous variables. The two subsamples differed significantly in educational level (χ^2^ = 11.88, *p* < 0.001).

#### 6.2.2 Item analysis results

In the item analysis of the 42-item initial HLC-N, all items met the retention criteria. The critical ratio (CR) values ranged from 9.01 to 19.70 (all > 3, *p* < 0.001), and the item-total correlation coefficients ranged from 0.528 to 0.810 (all > 0.4). No items were deleted based on the homogeneity test, as the Cronbach’s α coefficient of the total scale (0.977) did not increase after deletion of any single item (see Supplementary Table 1).

#### 6.2.3 Content validity results

Based on the evaluations of 16 Delphi experts on the relevance of the questionnaire items, the I-CVI of the initial 42-item HLC-N ranged from 0.813 to 1.00. The S-CVI/Ave for the 39-item scale was 0.952, exceeding the recommended threshold of 0.90 (21).

#### 6.2.4 Construct validity results

Exploratory factor analysis (EFA): EFA was performed sequentially on the calibration sample (*n* = 467). In the first EFA of the initial 42-item HLC-N, the KMO measure was 0.960, and Bartlett’s test of sphericity was significant (*χ^2^* = 18589.002, *p* < 0.01), indicating suitability for factor analysis. Three items (T1, M1, B8) had factor loadings below 0.50 and were deleted. No cross-loadings were identified.

A second EFA was conducted on the remaining 39 items. The KMO value was 0.958, and Bartlett’s test was significant (*χ^2^* = 17129.998, *p* < 0.01). Six factors with eigenvalues > 1 were extracted, explaining 72.558% of the variance, and all 39 items had factor loadings exceeding 0.50 (range: 0.511–0.855) (see Table 2). The scree plot showed the slope became gradual after the sixth factor (see Figure 1). These six factors represent five conceptual dimensions, as the Health Literacy Knowledge dimension was further split into two sub-factors: Policy and Regulation (3 items: K1, K5, K7) and Factors and Outcomes (4 items: K2, K3, K4, K6). The other four factors correspond directly to the remaining dimensions: Inter-professional and Resource Collaboration Competency (10 items), Health Education Communication Competency (8 items), Attitude and Motivation (6 items), and Health Education Practice Competency (8 items). The factor structure was consistent with the theoretical framework.

**Table 2:** Exploratory factor analysis: factor loadings of 39 items.

Item	Factor loading					
	1	2	3	4	5	6
T10	0.855					
T9	0.850					
T8	0.836					
T7	0.768					
T11	0.762					
T4	0.739					
T3	0.697					
T2	0.669					
T6	0.661					
T5	0.656					
B2		0.701				
B4		0.683				
B1		0.661				
B5		0.648				
B3		0.602				
B6		0.600				
B7		0.523				
B9		0.515				
M4			0.791			
M7			0.745			
M5			0.739			
M3			0.692			
M2			0.675			
M6			0.660			
P6				0.700		
P7				0.700		
P5				0.627		
P4				0.544		
P8				0.540		
P2				0.527		
P3				0.517		
P1				0.511		
K6					0.743	
K3					0.737	
K4					0.716	
K2					0.608	
K1						0.748
K7						0.731
K5						0.707
Eigenvalue	19.767	3.390	1.589	1.431	1.112	1.009
Variance explained (%)	50.684	8.692	4.075	3.669	5.865	5.587
Cumulative variance (%)	50.684	59.376	63.451	67.120	69.971	72.558
Cronbach’s alpha	0.955	0.921	0.922	0.937	0.853	0.831

**Figure 1:**
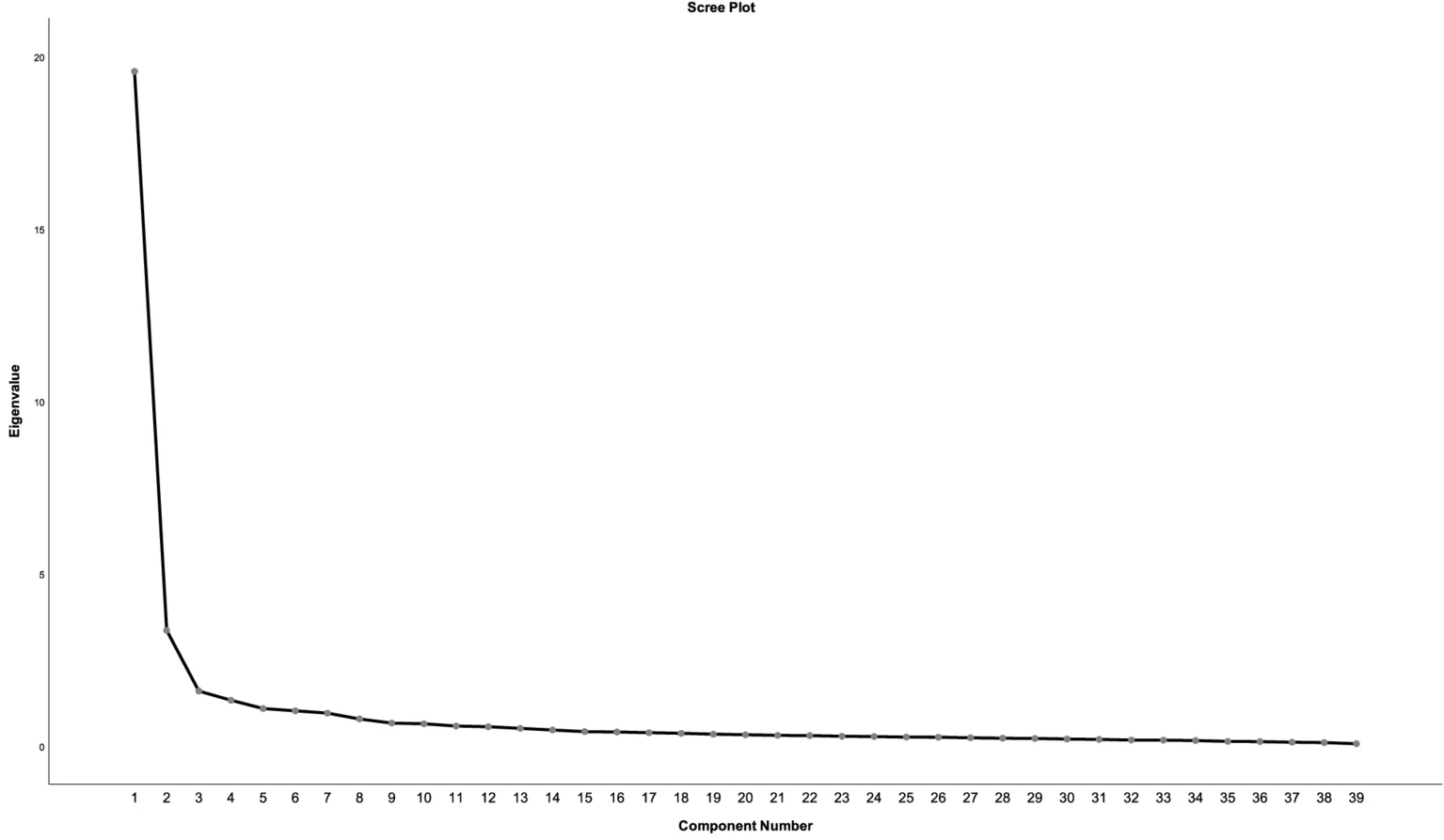
Scree plot of exploratory factor analysis.

Confirmatory factor analysis (CFA): CFA was conducted on the validation sample (*n* = 393) to test the six-factor structure of the 39-item HLC-N. As shown in Table 3, the model demonstrated good fit: *χ^2^/df* = 2.223 (< 3.0), RMSEA = 0.056 (< 0.08), CFI = 0.942, NFI = 0.900, IFI = 0.942, TLI = 0.937 (all > 0.90), and GFI = 0.830 (> 0.80). The standardized factor loadings and model structure are presented in Figure 2. These findings indicated that the six-factor model had sound structural stability (19).

**Table 3:** Confirmatory factor analysis: model fit indices.

Index	Recommended value	Acceptable value	Model result
χ^2^	—	—	1526.978
χ^2^/df	< 3.0	≤ 5.0	2.223
RMSEA	< 0.08	≤ 0.10	0.056
CFI	> 0.90	≥ 0.80	0.942
GFI	> 0.90	≥ 0.80	0.830
NFI	> 0.90	≥ 0.80	0.900
IFI	> 0.90	≥ 0.80	0.942
TLI	> 0.90	≥ 0.90	0.937

χ^2^/df, Chi-square/degrees of freedom ratio; RMSEA, Root mean square error of approximation; CFI, Comparative fit index; GFI, Goodness-of-fit index; NFI, Normed fit index; IFI, Incremental fit index; TLI, Tucker-Lewis index.

**Figure 2:**
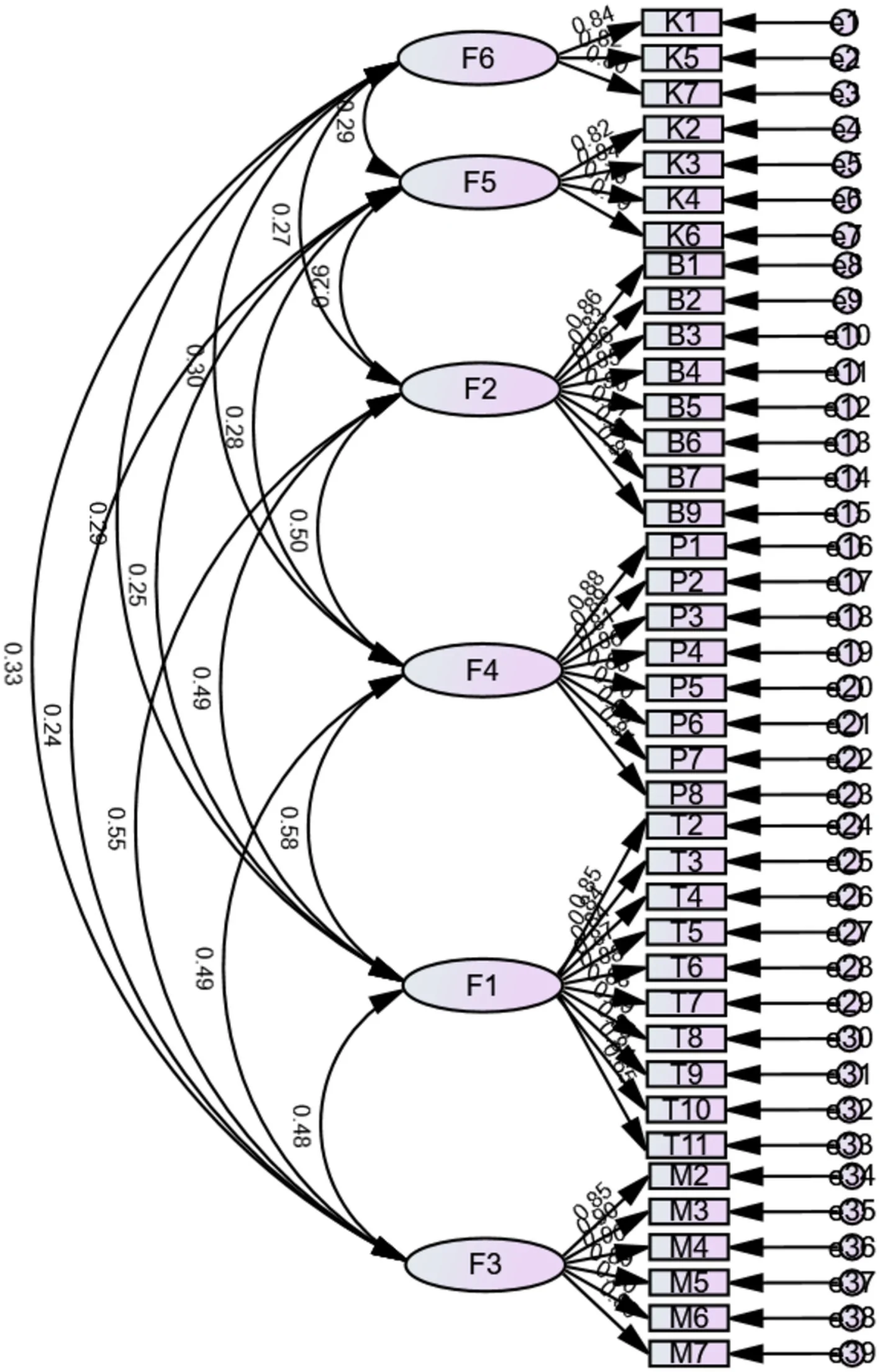
Path diagram of confirmatory factor analysis (six-factor model).

Convergent and discriminant validity: As Table 4 illustrates, all factor loadings exceeded 0.50 (0.645–0.904), CR (0.855–0.959) and AVE (0.658–0.791) met recommended thresholds (> 0.70 and > 0.50, respectively), and the square roots of AVE (0.811–0.889) exceeded inter-construct correlations (0.218–0.549), indicating good convergent and discriminant validity (19).

**Table 4:** Factor loadings, convergent validity, and discriminant validity matrix (Fornell-Larcker criterion).

Construct/item	λ	CR	AVE	P&R	F&O	HECL	HEPC	I&RCL	A&M
Policy & regulation		0.860	0.673	**0.820**					
K1	0.838								
K5	0.819								
K7	0.803								
Factors & outcomes		0.885	0.658	0.255**	**0.811**				
K2	0.822								
K3	0.843								
K4	0.792								
K6	0.787								
Health education communication competency		0.956	0.731	0.241**	0.239**	**0.855**			
B1	0.864								
B2	0.833								
B3	0.861								
B4	0.849								
B5	0.897								
B6	0.869								
B7	0.836								
B9	0.828								
Health education practice competency		0.948	0.698	0.260**	0.252**	0.470**	**0.835**		
P1	0.880								
P2	0.895								
P3	0.810								
P4	0.861								
P5	0.851								
P6	0.903								
P7	0.645								
P8	0.808								
Interprofessional & resource collaboration literacy		0.959	0.704	0.261**	0.236**	0.455**	0.549**	**0.839**	
T2	0.853								
T3	0.839								
T4	0.836								
T5	0.870								
T6	0.875								
T7	0.878								
T8	0.885								
T9	0.832								
T10	0.842								
T11	0.654								
Attitude & motivation		0.958	0.791	0.304**	0.218**	0.531**	0.459**	0.444**	**0.889**
M2	0.845								
M3	0.896								
M4	0.898								
M5	0.889								
M6	0.904								
M7	0.903								

Values on the diagonal (in bold) are the square roots of the Average Variance Extracted (AVE); off-diagonal values are Pearson correlation coefficients among constructs, **p < 0.01.

#### 6.2.5 Reliability results

As shown in Table 5, the HLC-N demonstrated good internal consistency. Cronbach’s α coefficients ranged from 0.820 to 0.958 for the five dimensions, with a total scale α of 0.958. Split-half reliability for the total scale was 0.827 (dimensions: 0.792–0.956). Test–retest reliability for the total scale was 0.927 (dimensions: 0.622–0.758), all exceeding or approaching the recommended threshold of 0.7–0.8 (23).

**Table 5:** Reliability of the Health Literacy Competencies Scale for nurses.

Dimension	Cronbach’s α (*n* = 393)	Split-half reliability (*n* = 393)	Test–retest reliability (*n* = 81)
Health Literacy Knowledge	0.820	0.792	0.632
Health Education Communication Literacy	0.956	0.934	0.742
Health Education Practice Competence	0.945	0.938	0.758
Interprofessional & resource collaboration literacy	0.958	0.931	0.743
Attitude & Motivation	0.958	0.956	0.622
Total scale	0.958	0.827	0.927

Split-half reliability was calculated using the Spearman-Brown coefficient.

### 6.3 Part 3: cross-sectional survey results

Participant characteristics: Of 680 nurses surveyed, 647 (95.15%) were included. As shown in Table 6, most were female (98.45%), aged 25–35 years (52.70%), with 5–15 years of experience (57.50%). Nearly 50% had no health literacy training.

**Table 6:** Sociodemographic characteristics of the participants *N* = 647.

Characteristic	Category	*n*	Percentage (%)
Gender	Male	10	1.55
	Female	637	98.45
Age (years)	≤24	66	10.20
	25–34	341	52.70
	35–45	200	30.91
	≥45	40	6.18
Professional title	Junior	283	43.74
	Intermediate	334	51.62
	Senior	30	4.64
Job category	Clinical nursing	537	83.00
	Nursing management	68	10.51
	Nursing support	11	1.70
	Others	31	4.79
Years of working	≤4	116	17.93
	5–15	372	57.50
	15–25	123	19.01
	≥25	36	5.56
Department	Internal medicine	299	46.21
	Surgery	217	33.54
	Obstetrics & Gynecology	56	8.66
	Pediatrics	55	8.50
	Others	20	3.09
Hospital level	Tertiary	487	75.27
	Secondary	93	14.37
	Primary	67	10.40
Education level	Associate degree	117	18.08
	Bachelor’s degree	521	80.53
	Master’s degree or above	9	1.39
Employment type	Permanent staff	136	21.02
	Agency-employed	64	9.89
	Contract staff	447	69.09
Monthly income (RMB)	≤4,999	280	43.28
	5,000–8,000	324	50.08
	≥8,001	43	6.65
Marital status	Unmarried	165	25.50
	Married	464	71.72
	Divorced	18	2.78
Health status	Good	433	66.92
	Fair	206	31.84
	Poor	8	1.24
Received health literacy training	Yes	361	55.80
	No	286	44.20
Received competency training	Yes	340	52.55
	No	307	47.45

*N* = 647. “Others” in department category includes ICU, Emergency Department, Outpatient Department, and Operating Room. “Agency-employed” refers to nurses dispatched through third-party human resource agencies.

HLC scores: The mean HLC score was 154.41 ± 20.60 (moderately high). The highest mean item score was Attitude and Motivation (4.17), and the lowest was Inter-professional and Resource Collaboration Competency (3.80), followed by Health Literacy Knowledge (3.89) (see Table 7).

**Table 7:** Scores of the Health Literacy Competencies Scale for nurses *N* = 647.

Dimension	No. of items	Possible range	Dimension score ( $\bar{x}\pm s$ )	Item mean score ( $\bar{x}\pm s$ )
Health Literacy Knowledge	7	7–35	27.24 ± 4.32	3.89 ± 0.62
Health Education Communication Literacy	8	8–40	32.89 ± 4.16	4.11 ± 0.52
Health Education Practice Competence	8	8–40	31.25 ± 4.82	3.91 ± 0.60
Interprofessional & Resource Collaboration Literacy	10	10–50	38.00 ± 6.66	3.80 ± 0.67
Attitude & Motivation	6	6–30	25.03 ± 3.19	4.17 ± 0.53
Total Scale	39	39–195	154.41 ± 20.60	3.96 ± 0.53

Item mean score = dimension total score/number of items. Grading criteria: 39–78 = low, 79–116 = moderate, 117–156 = upper-intermediate, 157–195 = high.

Univariate analysis: HLC scores differed significantly by health status (*F* = 5.865, *p* = 0.003), prior health literacy training (*t* = 6.714, *p* < 0.001), and prior competency training (*t* = 6.178, *p* < 0.001). No other variables were significant.

Multivariate analysis: Regression analysis (*F* = 6.456, *p* < 0.001) identified three independent predictors: health status (*β* = 0.087, *p* = 0.024), health literacy training (*β* = 0.164, *p* < 0.001), and competency training (*β* = 0.122, *p* = 0.012), explaining 7.8% of the variance (adjusted *R^2^* = 0.078). Health status was dichotomized for regression analysis due to the small number of participants in the ‘Poor’ category (n = 8). The variable was coded as Good = 1 and Fair/Poor = 0, with Fair/Poor serving as the reference group (see Table 8).

**Table 8:** Regression analysis of factors influencing health literacy competency *N* = 647.

Predictors	B (SE)	Beta	t	*p*	95% CI	VIF
(Constant)	146.389 (2.710)		54.020	<0.001	[141.068, 151.711]	
Intermediate^a^	−1.828 (1.906)	−0.044	−0.959	0.338	[−5.571, 1.916]	1.500
Senior or Above^a^	−3.502 (4.490)	−0.036	−0.780	0.436	[−12.319, 5.316]	1.474
6–15 years^b^	−3.190 (2.410)	−0.077	−1.323	0.186	[−7.924, 1.543]	2.347
16–25 years^b^	−1.753 (3.007)	−0.033	−0.583	0.560	[−7.658, 4.153]	2.301
26 + years^b^	−1.947 (4.323)	−0.022	−0.450	0.653	[−10.435, 6.541]	1.623
Bachelor’s Degree^c^	2.796 (2.268)	0.054	1.233	0.218	[−1.658, 7.249]	1.333
Master’s or Above^c^	12.083 (7.020)	0.069	1.721	0.086	[−1.703, 25.868]	1.117
Health Status^d^	3.792 (1.679)	0.087	2.258	**0.024**	[0.494, 7.089]	1.032
Health Literacy Training Exp.^e^	6.817 (1.995)	0.164	3.417	**0.001**	[2.899, 10.735]	1.623
Competency Training Exp.^e^	5.021 (1.996)	0.122	2.515	**0.012**	[1.101, 8.940]	1.642

Dependent variable: Health Literacy Competency. Model fit: R^2^ = 0.092, Adjusted R^2^ = 0.078, F (10, 636) = 6.456, *p* < 0.001, Durbin-Watson = 1.850. Reference groups: ^a^Junior or below title; ^b^ ≤ 5 years; ^c^Associate degree or below; ^d^Fair/Poor (Good = 1, Fair/Poor = 0); ^e^No training experience. Bold values indicate statistical significance (*p* < 0.05).

## 7 Discussion

### 7.1 Scale development and psychometric evaluation

#### 7.1.1 Scientific rationale of the scale

This study employed the Iceberg Model from competency theory as the theoretical framework to construct the HLC development model for nurses. This theoretical grounding addresses a key limitation of existing tools, which often lack explicit theoretical foundations (15, 17). Through literature review and semi-structured interviews with nurses, a 53-item Delphi consultation version of the HLC-N was developed. A two-round Delphi expert consultation was conducted to systematically integrate the professional opinions of senior experts in the fields of HLC and scale development. All consulted experts had more than 10 years of work experience, and the expert authority coefficients were all above 0.8 (*Cr* = 0.880). The Kendall’s W increased from 0.217 in the first round to 0.355 in the second round (*p* < 0.05), indicating improved expert consensus (23).

After the two rounds of Delphi consultation, items in the scale pool were revised based on expert opinions to ensure accurate wording, clear content, and logical item sequencing, resulting in a 42-item initial version of the HLC-N. A pilot test with 30 nurses confirmed that the initial scale items were clearly worded, well-understood, and convenient for nurses to complete.

During the item analysis phase, the critical ratio method, correlation coefficient method, and homogeneity test were used to screen items, followed by EFA for factor extraction, during which three items with factor loadings below 0.5 were deleted. CFA was then used to validate the stability of the 39-item scale’s dimensional structure, and reliability testing ultimately confirmed the final version of the HLC-N, comprising 5 dimensions and 39 items.

#### 7.1.2 Reliability of the scale

The Cronbach’s α coefficients for the total scale (0.958) and all dimensions (0.820–0.958) exceeded the recommended threshold of 0.8 for the total scale and 0.6 for subscales (23), indicating good internal consistency. The split-half reliability of the total scale was 0.827, with dimension coefficients ranging from 0.792 to 0.956. The slightly lower split-half reliability for the Health Literacy Knowledge dimension (0.792) may be attributed to its two-factor structure (“Policy and Regulation” and “Factors and Outcomes”), as the distribution of items during split-half may affect the correlation between the two halves. Given the satisfactory Cronbach’s α for this dimension (0.820) and its sub-factors (0.859, 0.855), the overall reliability was considered acceptable.

The test–retest reliability coefficient for the total scale was 0.927, indicating good temporal stability. The lower coefficients for the Attitude and Motivation (0.622) and Health Literacy Knowledge (0.632) dimensions require consideration. Nurses’ professional attitudes and work motivation are susceptible to recent work pressure, job burnout, or policy changes, which may fluctuate over a two-week period (28). Similarly, knowledge-based scales may be affected by natural forgetting or new learning between measurements. These findings align with those reported in similar scale development studies, where attitude and knowledge dimensions typically show lower test–retest reliability than skill-based dimensions (23).

#### 7.1.3 Validity of the scale

The content validity of the HLC-N was supported by I-CVI values (0.813–1.000) and an S-CVI/Ave of 0.952, exceeding the recommended standard of 0.90 (21). The EFA extracted six factors with eigenvalues > 1, explaining 72.558% of the total variance, which exceeds the recommended threshold of 50% (18). All factor loadings exceeded 0.5, with no cross-loadings, indicating a clean factor structure consistent with the theoretical framework.

The CFA results demonstrated good model fit: *χ^2^/df* = 2.223 (< 3.0), RMSEA = 0.056 (< 0.08), CFI = 0.942, NFI = 0.900, IFI = 0.942, TLI = 0.937 (all > 0.90), and GFI = 0.830 (> 0.80). While GFI was slightly below the ideal standard of 0.90, this is acceptable for models with complex structures and sample sizes around 400 (19). All other fit indices met or exceeded recommended thresholds, supporting the construct validity of the six-factor model.

Convergent validity was supported by AVE values (0.658–0.791 > 0.5) and CR values (0.855–0.959 > 0.7) for all dimensions (29). Discriminant validity was confirmed as the square root of AVE for each dimension exceeded its correlations with other dimensions (0.218–0.549). These psychometric properties compare favorably with existing HLC tools such as the IOHLC (13), which reported AVE values of 0.50–0.68 and CR values of 0.82–0.91, and the scale developed by Feng et al. (16), which reported AVE values of 0.55–0.72.

#### 7.1.4 Importance of scale development

The HLC-N addresses critical gaps in existing HLC assessment tools. Unlike the IOHLC (13), which targets generalized health professionals, the HLC-N is specifically designed for nurses, with items reflecting clinical practice scenarios such as continuous health education throughout hospitalization, emotional support, and trust-building. Unlike instruments focused solely on “health education ability” (14, 15) the HLC-N encompasses a broader range of competencies including inter-professional collaboration, resource navigation, and intrinsic motivation to improve patient HL.

The five-dimensional structure of the HLC-N corresponds to the hierarchical levels of the Iceberg Model (knowledge → skills → self-concept → traits → motivation), providing a theoretically grounded and comprehensive assessment of nurses’ HLC. The scale’s cross-scenario applicability—covering primary, secondary, and tertiary hospitals as well as multiple clinical departments—addresses the limited generalizability of existing tools restricted to community (15) or dental (16) settings. These features make the HLC-N a promising tool for both research and clinical practice, enabling the assessment of nurses’ HLC and the evaluation of targeted training interventions.

### 7.2 Current status and influencing factors of nurses’ HLC

#### 7.2.1 General characteristics of the participants

The demographic characteristics of the sample (98.45% female, 52.70% aged 25–35 years, 57.50% with 5–15 years of experience) are consistent with the Chinese nursing workforce profile (30), supporting the representativeness of the sample. Notably, nearly 50% of participants had not received health literacy or competency-related training. Although this represents an improvement from the 34.5% training participation rate reported by Congying et al. (31), it remains concerning given that training experience is a strong predictor of HLC (32). This finding underscores the urgent need to expand training coverage before optimizing training content.

#### 7.2.2 Nurses’ HLC at a moderately high level with weak knowledge and collaboration competencies

The mean HLC score (154.41 ± 20.60) indicates a moderately high level, consistent with findings from Guizhou Province (33) and higher than those from Sichuan Province (34). The dimension-specific scores revealed the highest performance in Attitude and Motivation (4.17), reflecting nurses’ intrinsic drive to improve patient HL. However, the relatively lower scores in Health Literacy Knowledge (3.89) and Inter-professional and Resource Collaboration Competency (3.80) highlight areas requiring targeted intervention.

The deficiency in HL knowledge is particularly concerning, as it may limit nurses’ ability to effectively educate patients and navigate complex health information systems. Similar knowledge gaps have been reported among medical staff in China (33, 35) and among resident physicians in the United States (36). The weakness in inter-professional collaboration competency is consistent with findings from a multinational study in which nurses scored lowest on the “teamwork” dimension of patient safety competency (37). This suggests that inter-professional education and team communication training should be prioritized.

The finding that health status independently influences HLC (*β* = 0.087, *p* = 0.024) aligns with previous research (33). Nurses in better health may have more energy and motivation to engage in HL promotion activities and may be more likely to acquire health knowledge for personal use, which translates into professional competency.

The finding that training experience independently influences HLC (β = 0.122, *p* = 0.012) aligns with previous research (33, 34). Liu et al. (34), for instance, reported a β of 0.112 for competency-related training. This finding supports the effectiveness of training interventions and highlights the need to ensure that more nurses have access to systematic HL education.

## 8 Limitations

Several limitations should be acknowledged. First, convenience sampling for psychometric validation and the geographical restriction to Chongqing for the cross-sectional survey may limit generalisability. Second, the cross-sectional design precludes causal inferences. Third, the EFA and CFA subsamples differed significantly in educational background (*χ^2^* = 11.88, *p* < 0.001), potentially introducing bias. Fourth, the hierarchical pathways of the Iceberg Model were not empirically tested. Future research should adopt stratified sampling, expand geographical coverage, employ longitudinal designs, and test theoretical relationships using structural equation modeling.

## 9 Conclusion

The HLC-N is a reliable and valid 39-item, five-dimensional scale for assessing nurses’ health literacy competencies in China. Nurses in Chongqing general hospitals demonstrated moderately high HLC (154.41 ± 20.60), with positive attitudes but weaker knowledge and collaboration competencies. Health status and training experience were independent influencing factors, and nearly 50% had never received relevant training. Hospital administrators should prioritize HLC assessment and develop targeted training programs focusing on health literacy knowledge and inter-professional collaboration.

### 9.1 Practice implications

The HLC-N may serve as a reference for the theoretical and in-service training of nurses’ health literacy competencies. Hospital administrators can use this tool to regularly assess nurses’ HLC and identify those with insufficient competencies. Targeted training programs should focus on health literacy knowledge and inter-professional collaboration. Expanding training coverage is the primary task, followed by optimizing training content and methods. Future studies should investigate different nursing groups to further validate the scale’s applicability across diverse patient education contexts.

## Data Availability

The original contributions presented in the study are included in the article/Supplementary material, further inquiries can be directed to the corresponding author.
