# Supplementary material for: Development and psychometric validation of a health literacy competency scale for nurses, and its application in a cross-sectional survey: a methodological study
Source: Front Public Health. 2026 Sep 28;14:1978033. doi: 10.3389/fpubh.2026.1978033 (PMC13655236; doi:10.3389/fpubh.2026.1978033)
Supplement: Supplementary file 1 [file Table_1.docx]

**Supplementary File**

Table 1 Item Analysis Results of the 42-item scale *N*=467

| **item** | **CR** | ***r*** | **Cronbach’s α** | **I-CVI** |
| --- | --- | --- | --- | --- |
| K1 | 10.65 | 0.558 | 0.975 | 0.813 |
| K2 | 9.01 | 0.528 | 0.975 | 1.000 |
| K3 | 10.46 | 0.606 | 0.975 | 1.000 |
| K4 | 10.59 | 0.590 | 0.975 | 0.938 |
| K5 | 11.41 | 0.629 | 0.975 | 1.000 |
| K6 | 9.77 | 0.609 | 0.975 | 1.000 |
| K7 | 13.99 | 0.671 | 0.975 | 0.813 |
| B1 | 11.62 | 0.687 | 0.974 | 1.000 |
| B2 | 11.70 | 0.702 | 0.974 | 1.000 |
| B3 | 16.68 | 0.751 | 0.974 | 0.938 |
| B4 | 12.02 | 0.627 | 0.975 | 0.938 |
| B5 | 13.30 | 0.702 | 0.974 | 0.938 |
| B6 | 15.10 | 0.738 | 0.974 | 0.938 |
| B7 | 16.78 | 0.753 | 0.974 | 1.000 |
| B8 | 14.92 | 0.753 | 0.974 | 1.000 |
| B9 | 15.41 | 0.752 | 0.974 | 0.875 |
| P1 | 16.81 | 0.754 | 0.974 | 1.000 |
| P2 | 16.61 | 0.804 | 0.974 | 1.000 |
| P3 | 16.91 | 0.786 | 0.974 | 0.938 |
| P4 | 15.02 | 0.777 | 0.974 | 1.000 |
| P5 | 17.85 | 0.798 | 0.974 | 1.000 |
| P6 | 17.21 | 0.810 | 0.974 | 1.000 |
| P7 | 10.29 | 0.626 | 0.975 | 0.813 |
| P8 | 17.15 | 0.803 | 0.974 | 1.000 |
| T1 | 15.74 | 0.778 | 0.974 | 1.000 |
| T2 | 17.41 | 0.758 | 0.974 | 0.938 |
| T3 | 17.56 | 0.731 | 0.974 | 0.938 |
| T4 | 19.36 | 0.754 | 0.974 | 1.000 |
| T5 | 17.96 | 0.795 | 0.974 | 1.000 |
| T6 | 17.16 | 0.810 | 0.974 | 1.000 |
| T7 | 16.38 | 0.733 | 0.974 | 1.000 |
| T8 | 18.76 | 0.754 | 0.974 | 1.000 |
| T9 | 19.70 | 0.740 | 0.974 | 0.938 |
| T10 | 16.24 | 0.708 | 0.974 | 0.938 |
| T11 | 12.03 | 0.625 | 0.975 | 0.813 |
| M1 | 12.34 | 0.681 | 0.975 | 0.875 |
| M2 | 11.61 | 0.646 | 0.975 | 1.000 |
| M3 | 11.72 | 0.671 | 0.975 | 1.000 |
| M4 | 10.73 | 0.637 | 0.975 | 0.875 |
| M5 | 12.14 | 0.682 | 0.975 | 1.000 |
| M6 | 13.55 | 0.737 | 0.974 | 0.813 |
| M7 | 13.70 | 0.733 | 0.974 | 0.938 |
